# Polarity-Sensitive Probes for Superresolution Stimulated Emission Depletion Microscopy

**DOI:** 10.1016/j.bpj.2017.06.050

**Published:** 2017-07-19

**Authors:** Erdinc Sezgin, Falk Schneider, Victoria Zilles, Iztok Urbančič, Esther Garcia, Dominic Waithe, Andrey S. Klymchenko, Christian Eggeling

**Affiliations:** 1 MRC Human Immunology UnitWeatherall Institute of Molecular Medicine, University of Oxford, Oxford, United Kingdom; 2 Wolfson Imaging Centre Oxford, Weatherall Institute of Molecular Medicine, University of Oxford, Oxford, United Kingdom; 3 CNRS UMR 7213, Laboratoire de Biophotonique et Pharmacologie, University of Strasbourg, Illkirch Cedex, France

## Abstract

The lateral organization of molecules in the cellular plasma membrane plays an important role in cellular signaling. A critical parameter for membrane molecular organization is how the membrane lipids are packed. Polarity-sensitive dyes are powerful tools to characterize such lipid membrane order, employing, for example, confocal and two-photon microscopy. The investigation of potential nanodomains, however, requires the use of superresolution microscopy. Here, we test the performance of the polarity-sensitive membrane dyes Di-4-ANEPPDHQ, Di-4-AN(F)EPPTEA, and NR12S in superresolution stimulated emission depletion microscopy. Measurements on cell-derived membrane vesicles, in the plasma membrane of live cells, and on single virus particles, show the high potential of these dyes for probing nanoscale membrane heterogeneity.

## Introduction

The lateral organization of molecules in the cellular plasma membrane has significant influence on cellular functions. For example, lipid-lipid and lipid-protein interactions facilitate the segregation of plasma membrane molecules into clusters or nanodomains that constitute catalytic platforms for a myriad of activities such as cellular signaling (1, 2). Lateral heterogeneity in lipid order, i.e., how the packing of lipids varies over space, and how this is involved in molecular segregation, is particularly of interest because membrane order may modulate protein functionality (3, 4, 5, 6, 7). Saturated lipids can be packed relatively more tightly and form ordered membrane environments in contrast to unsaturated lipids that yield less ordered (i.e., more disordered) membranes. Certain lipid combinations (especially those involving cholesterol) result in macroscopic phase separation in model membranes, which can readily be resolved by confocal microscopy (8). Similar segregation has also been observed in vesicles generated from plasma membrane (9). In living cells, however, heterogeneities in lipid properties supposedly occur only on a subdiffraction spatial scale, which has been inferred from indirect measurements. Clearly, methodological advances are needed to adequately tackle this phenomenon of high biological relevance.

**Figure 1:**
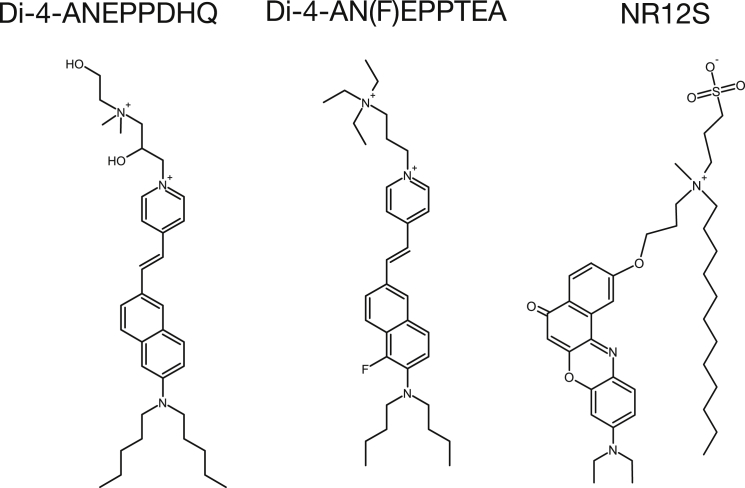
Structures of the polarity-sensitive membrane dyes employed in this study: Di-4-ANEPPDHQ, Di-4-AN(F)EPPTEA, and NR12S.

Polarity-sensitive fluorescent probes such as Laurdan are useful tools to study lipid order (10, 11, 12). These dyes change their emission spectrum depending on the environmental conditions; for example, they exhibit a relatively more red-shifted fluorescence spectrum in more polar solvents (13, 14). More tightly packed (ordered) lipid environments accommodate a lower number of water molecules and are therefore relatively less polar than disordered environments (15). In turn, the fluorescence emission spectrum of the polarity-sensitive dyes is more blue shifted in ordered membrane environments. This shift can be used to quantify the molecular ordering and to visualize lateral heterogeneity in cellular membranes (16). These probes have been used in combination with confocal or multiphoton microscopy (17, 18, 19, 20); however, the diffraction-limited spatial resolution of these techniques does not allow observation and full characterization of nanodomains or clusters in the plasma membrane. A remedy to this limitation is superresolution optical microscopy, such as stimulated emission depletion (STED) (21, 22). Although STED microscopy has recently been employed to study nanoscale plasma membrane organization (23, 24, 25, 26, 27), the combination of this technique with polarity-sensitive probes has not yet been realized. Traditional polarity-sensitive probes such as Laurdan and Prodan already suffer from their near-UV excitation and low photostability in conventional microscopy (17). Recently developed probes such as Di-4-ANEPPDHQ (28), Di-4-AN(F)EPPTEA (29), and NR12S (30) (Fig. 1) show higher brightness and a red-shifted fluorescence emission. Thus, these probes may be suitable for imaging on a STED microscopy setup. Here, we test the performance of these polarity-sensitive membrane probes in STED microscopy. Using cell-derived membrane vesicles, live cells, and HIV particles, we highlight their potential for studying lateral differences in plasma membrane order with subdiffraction spatial resolution.

## Materials and Methods

### Probes

Laurdan and Di-4-ANEPPDHQ were purchased from Thermo Fisher Scientific (Waltham, MA). Di-4-AN(F)EPPTEA (29) was a kind gift from Prof. Leslie Loew. F2N12SM, FC12SM, PA, and NR12S were made as described in the literature (30, 31, 32, 33). Abberior Star Red (formerly known as KK114)-labeled 1,2-dipalmitoyl-*sn*-glycero-3-phosphoethanolamine (DPPE) was obtained from Abberior (Göttingen, Germany).

### Preparation of cells and giant plasma membrane vesicles

CHO cells were maintained in F12/DMEM (Sigma-Aldrich, St. Louis, MO) medium supplemented with 10% FBS (Sigma-Aldrich) and 1% L-glutamine (Sigma-Aldrich). Cells were seeded on 35 mM petri dishes (plastic dishes for GPMV experiments (Nunc, Roskilde, Denmark), and glass-bottom dishes (Ibidi, Fitchburg, WI) for cellular imaging experiments) for three days before the experiments. For green fluorescent protein (GFP) transfection, the cells were transfected with cytoplasmic GFP plasmid in the second day using Turbofect (Thermo Fisher Scientific) transfection agent according to the manufacturer’s protocol. GPMVs were prepared using 25 mM paraformaldehyde (Sigma-Aldrich) and 2, 5, or 30 mM Dithiothreitol (DTT; Sigma-Aldrich) in a giant plasma membrane vesicle (GPMV) buffer for non-phase-separated (2 mM DTT) and phase-separated (5 mM DTT for small domains and 30 mM DTT for large domains) vesicles, as detailed in Sezgin et al. (34). GPMVs were immobilized for imaging as shown in Fig. S1 and as detailed in Schneider et al. (35). Cells were labeled with the polarity-sensitive membrane probes or fluorescent lipid analogs at 1 *μ*g/mL final probe concentration in phosphate buffered saline (PBS) for 5 min before imaging. Live cell imaging was performed in L-15 medium (Sigma-Aldrich) without Phenol Red. GPMVs were labeled with 0.1–0.25 *μ*g/mL final probe concentration before imaging. This concentration range was previously found not to perturb the membranes notably (36). All imaging experiments were performed at room temperature.

### Preparation of virus particles

HIV virus particles were prepared as detailed in Chojnacki et al. (37). In brief, they were generated from the tissue culture supernatant of 293T cells cotransfected using polyethyleneimine with 14 *μ*g pCHIV per 10 cm dish. Tissue culture supernatants were harvested 48 h after transfection, cleared by filtration through a 0.45 *μ*m nitrocellulose filter, and particles were purified by ultracentrifugation through a 20% (w/w) sucrose cushion at 70,000 × *g* (average) for 2 h at 4°C. Virus-containing gradient fractions were diluted in PBS and pelleted at 70,000 × *g* (average) for 2 h at 4°C. Particles were resuspended in ice-cold 20 mM HEPES/PBS pH 7.4, snap frozen and stored in aliquots at −80°C. All ultracentrifugation steps were performed in SW 41 Ti rotor. For microscope measurements, purified virus particles were left to adhere to glass coverslips, coated with poly-L-lysine (Sigma-Aldrich), for 30 min. Coverslips were blocked using 2% bovine serum albumin (Sigma-Aldrich)/PBS for 15 min. Particles were incubated with polarity-sensitive dyes for 30 min at 10 *μ*g/mL final probe concentration. After staining, the particles were washed and mounted in PBS, followed by microscopy analysis. All steps were carried out at room temperature.

### Conventional spectral imaging

**Figure 2:**
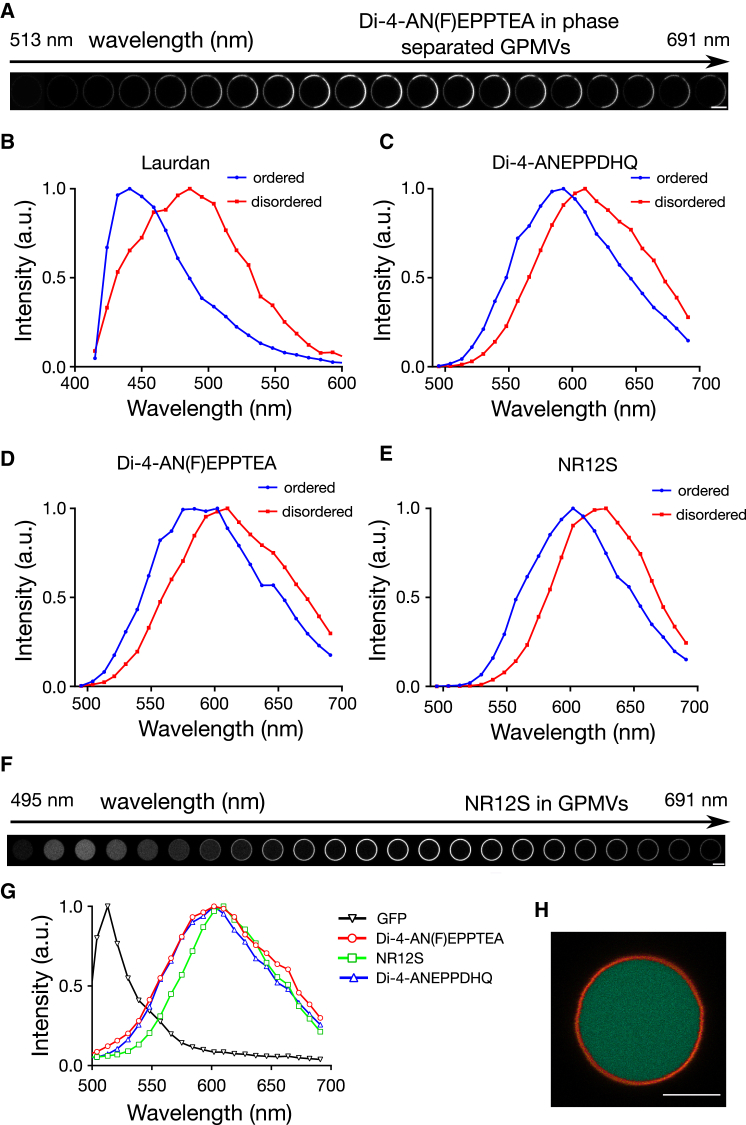
Confocal spectral imaging with the polarity-sensitive probes in CHO cell-derived GPMVs. (*A*) Given here are representative confocal images of the equatorial plane of a phase-separated GPMV stained with Di-4-AN(F)EPPTEA for subsequent 9-nm-wide spectral windows between 513 and 691 nm, indicating the emergence of shifted fluorescence maxima in different parts of the vesicle. (*B*–*E*) Shown here are normalized exemplary fluorescence emission spectra as determined from images as in (*A*) of (*B*) Laurdan, (*C*) Di-4-ANEPPDHQ, (*D*) Di-4-AN(F)EPPTEA, and (*E*) NR12S in the disordered (*red*) and ordered (*blue*) phases of the phase-separated GPMVs. (*F*) Given here are representative confocal images of the equatorial plane of a non-phase-separated GPMV derived from CHO cells transfected with cytoplasmic GFP and membrane stained with NR12S for subsequent 9-nm-wide spectral windows between 495 and 691 nm, indicating a spectral separation of fluorescence signal from the GPMV body (GFP) and membrane (NR12S). (*G*) Given here are normalized exemplary fluorescence emission spectra of GFP (*black*) and the polarity-sensitive dyes in non-phase-separated GPMVs (Di-4-ANEPPDHQ (*blue*), Di-4-AN(F)EPPTEA (*red*), and NR12S (*green*)). (*H*) Shown here is a fluorescence signal from (*F*) for the spectral ranges between 500 and 530 nm (*green*) and 570 to 691 nm (*red*), indicating clear separation of the GFP and NR12S signal, respectively. Scale bars represent 10 *μ*m. To see this figure in color, go online.

The conventional spectral imaging for Figs. 2, S3, and S4 was performed with an LSM 780 confocal microscope (Zeiss Microsystems, Mannheim, Germany) equipped with a 32-channel GaAsP detector array. Laser light at 405 nm was selected for fluorescence excitation of Laurdan and 488 nm for the other polarity-sensitive probes. The *λ*-detection range was set between 415 and 691 nm for Laurdan, and between 495 and 691 nm for the other dyes. The images were saved in .lsm file format and then analyzed with our freely available Fiji/ImageJ plugin (National Institutes of Health, Bethesda, MD), as described in Sezgin et al. (38).

### Confocal and STED imaging

All other images were acquired using a SP8 STED microscope (Leica Microsystems) equipped with a HC PL APO C S2 100×/1.40 oil objective, a pulsed (80 MHz) white light laser (WLL), and a pulsed 775 nm STED laser. For excitation, we selected 488 nm from the WLL. The master laser power for the WLL was 70 *μ*W and for the STED laser ∼280 mW (maximum power, measured at the back aperture of the objective). The laser light was further filtered using notch filters 488/561/633 nm (for the excitation) and 775 nm (for STED). Before each measurement session, the beam alignment of the WLL and the STED laser was checked. The alignment was performed with the 592 nm STED laser (reference laser). In the experiments, the power of the WLL laser was in the range of 10–25% of the master power, resulting in ∼10–20 *μ*W, whereas the employed STED laser power was ∼200 mW at the back aperture of the objective. The emission was detected using hybrid detectors. When two wavelength ranges were needed, 520–570- and 620–700-nm intervals were selected. Detector gating between 1.5 and 6 ns was applied. Two image sequences were taken: the first sequence without, and the second sequence with the STED laser on. The first sequence provided the standard confocal image whereas the second sequence provided the high-resolution STED microscopy image. The scanning speed was set to 1800 Hz line frequency and a line-averaging over four lines was applied. The pixel size was optimized for the STED microscopy image and kept the same for confocal (∼20 nm/pixel). With these settings, the acquisition of a 40 × 40-*μ*m image took ∼5 s.

### Calculation of membrane width from microscopy images

The recorded images were analyzed with the macro program “one line” in the software Fiji to determine the full width at half-maximum (FWHM) values of the intensity profiles across the membrane in the microscope images. The FWHM analysis was carried out for multiple intensity line profiles across the imaged membrane. A multiplexed FWHM measuring algorithm was designed and written using ImageJ macro language (https://github.com/dwaithe/generalMacros/tree/master/FWHM%20bulk%20measure). Using the ImageJ interface, the user defines a line using the “Segmented line” tool, which follows the contour of the vesicle to be measured. The algorithm then interpolates along the user-defined line and defines points at regular intervals (three-pixel gap) along the line. At each interpolated point on the line, perpendicular lines are drawn 40 pixels in length and centered on the interpolated line. Along each of these perpendicular lines the intensity values are sampled and a Gaussian curve is fit to the intensity line profiles using the ImageJ curve fitting plugin (see Fig. S2). The parameters of each curve are then output and the FWHM calculated for each curve (2√(2ln2)^∗^*σ*), where *σ* is the SD of the Gaussian fit. The extracted FWHM values were averaged over at least 10 images. Confocal and STED images were treated in the same way.

### Calculation of generalized polarization

Spectrally resolved images were taken with the same confocal/STED setup mentioned above. Ordered (lower wavelength) and disordered (higher wavelength) channels were set to 520–570 nm (*I*_1_) and 620–700 nm (*I*_2_), respectively. The confocal and STED images were taken sequentially. Generalized polarization (GP) maps (*I*_1_ – *I*_2_)/(*I*_1_ + *I*_2_) were created from these images using the freely available GP Plugin published before (38). For the images to be readable by the plugin, labels were assigned to the two channels with the command Image → Stacks → Tools → Set Label. These labels were automatically recognized by the plugin. Details on the plugin are outlined in Sezgin et al. (38).

### Comparison of photostability of different probes

For the comparison of probes’ photostability, live cells were labeled with the polarity-sensitive dyes or with KK114-labeled DPPE lipid analog as detailed in previous sections. Twenty sequential STED images of a 30 × 30-*μ*m area were taken without any time interval between them using 20 *μ*W excitation power and 200 mW STED power, with two-frame averaging and 512 × 512 pixels. For the quantification, the fluorescence emission signal was calculated from the portions of the membrane with ImageJ → Stacks-T Functions → Intensity v Time Monitor. Finally, the intensities were normalized to “1” for comparison.

## Results and Discussion

In our experience, a polarity-sensitive membrane probe adequate for advanced imaging (such as time-lapse or superresolution imaging) of lateral plasma membrane heterogeneity should have 1) a high sensitivity to membrane lipid packing, 2) a high photostability, and 3) an appropriately narrow emission spectrum to allow its combination with other fluorescent probes for labeling membrane proteins. Such properties have been indicated for recently developed probes such as Di-4-ANEPPDHQ, Di-4-AN(F)EPPTEA, and NR12S (17, 28, 29, 30, 32, 33) (Fig. 1).

We first compared the performance of the probes with respect to their sensitivity to differences in lipid order. For this purpose, we employed phase-separated model membranes that feature coexistence of a disordered and an ordered membrane phase. To mimic conditions in the plasma membrane as closely as possible, we used cell-derived GPMVs (9, 34, 39). The difference in lipid packing between disordered and ordered phases has been shown to be much lower and more physiological in GPMVs than in purely artificial phase-separated model membrane systems such as giant unilamellar vesicles (3, 40). Therefore, GPMVs are better tools to test the performance of the probes when their applicability in the cellular context is evaluated. Fig. 2 *A* shows confocal images, obtained in different spectral ranges by spectral imaging (38) of the equatorial plane of phase-separated GPMVs derived from the plasma membrane of CHO cells and labeled with the probe Di-4-AN(F)EPPTEA. At a first glance, a clear difference in intensity between different membrane phases of the GPMV within each single image in the spectral series can be observed, possibly arising from either nonequal partitioning of the probe into different membrane phases or altered excitation/emission properties in the different membrane environments (or both). More importantly, these images already indicate the differences in the emission spectra of the probes in the ordered and disordered phases, characteristic for polarity-sensitive dyes. This difference is further demonstrated in Fig. S3 by the calculation of the ratios of the fluorescence intensities detected in the ordered and disordered phases in each spectral channel.

For a better visualization and quantification of the spectral shifts, we extracted the fluorescence emission spectra of the investigated polarity-sensitive probes from the membrane segments in the ordered and disordered phases of GPMVs, with Laurdan as a well-known standard (Fig. 2, *B*–*E*). Although still lower than with Laurdan (∼50 nm, Fig. 2 *B*) (13), all three probes showed a clearly visible spectral shift of >30 nm between the peak positions in the ordered and disordered phases.

In contrast, other recently developed polarity-sensitive probes F2N12SM (32), FC12SM (32), and PA (33) showed much lower spectral shifts <15 nm (Fig. S4). We concluded that Di-4-ANEPPDHQ, Di-4-AN(F)EPPTEA, and NR12S are sensitive enough to distinguish ordered and disordered environments in cellular membranes.

Protein functionality may be modulated by the state of the lipid order of its immediate membrane environment (7, 41). Therefore, it is crucial to be able to determine lipid packing around specific proteins, which requires simultaneous labeling and observation of the proteins (e.g., using GFP) and of polarity-sensitive dyes. For distinguishing both, their spectra have to be well separated, i.e., the spectra of the polarity-sensitive dyes have to be narrow enough not to significantly overlap with the fluorescence emission spectrum of, for example, GFP. We therefore performed spectral imaging on non-phase-separated GPMVs derived from CHO cells transfected with cytoplasmic GFP (i.e., the GPMVs were filled with GFP) and membrane-labeled with the selected polarity-sensitive dyes. Here, we chose non-phase-separated GPMVs, because we now aimed at separating two homogeneous features (membranes and cytosol). Fig. 2 *F* shows confocal images for different spectral ranges of the equatorial plane of such a GPMV, indicating a clear spectral separation of fluorescence signal from the GPMV cytosol (GFP) and membrane (NR12S). The possibility of separating fluorescence emission from GFP and the polarity-sensitive probes is indicated in Fig. 2 *G*, which depicts the fluorescence emission spectra reconstructed from the spectral images of the GPMV bodies (GFP) and membranes (polarity-sensitive probes). In all cases (Di-4-ANEPPDHQ, Di-4-AN(F)EPPTEA, and NR12S), a significant separation between both spectra is observed. With appropriate filters (such as a 500–530-nm band-pass filter and a 570-nm long-pass filter for GFP and the polarity-sensitive dyes, respectively), the fluorescence signals from GFP and the polarity-sensitive dyes can be clearly separated as highlighted in Fig. 2 *H*, which plots the fluorescence signal from Fig. 2 *F* by recombining the spectral ranges between 500 and 530 nm (*green*) and 570–691 nm (*red*) into a two-channel image.

**Figure 3:**
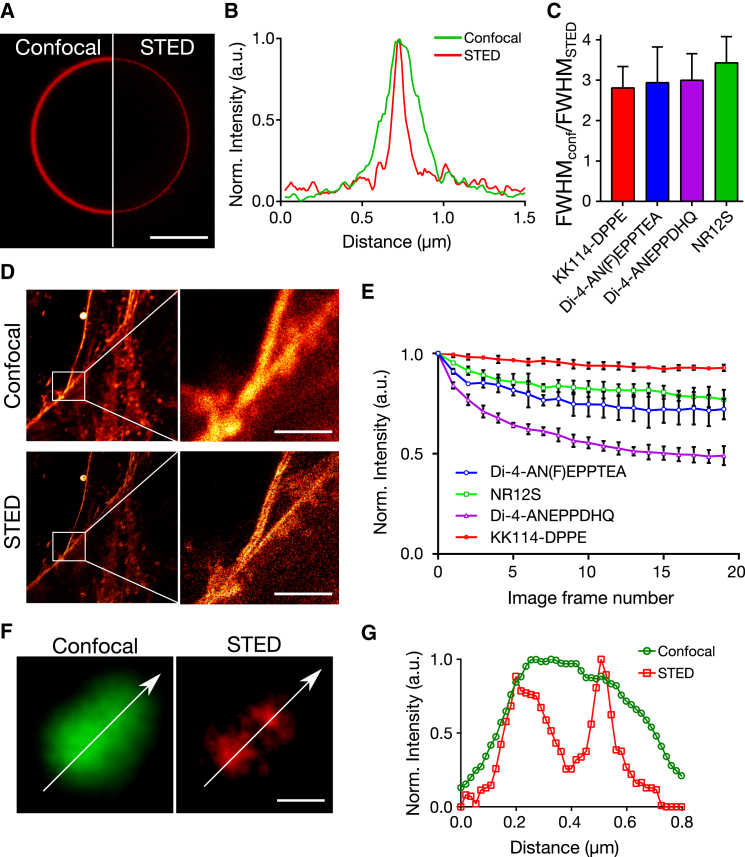
STED microscopy of polarity-sensitive membrane probes. (*A*) Given here are representative confocal (*left*) and STED (*right*) microscopy images of the equatorial plane of the same CHO cell-derived GPMV stained with Di-4-AN(F)EPPTEA. Scale bars represent 10 *μ*m. (*B*) Shown here are intensity line profiles through the membrane as obtained from the confocal (*green*) and STED (*red*) images of (*A*). (*C*) Given here is the average ratio of FWHM values obtained from the line profiles through the membranes in confocal and STED microscopy images of the CHO cell-derived GPMVs stained with Di-4-ANEPPDHQ (*purple*), Di-4-AN(F)EPPTEA (*blue*), NR12S (*green*), and a KK114-labeled phospholipid (*red*). Error bars are SD from at least 10 measurements. (*D*) Given here are representative confocal (*upper*) and STED (*lower*) microscopy images of the same CHO cells labeled with Di-4-AN(F)EPPTEA (*left*, overviews; *right*, closeups into marked areas), highlighting the improvement in spatial resolution by separation of the plasma membranes of two adjacent cells. Scale bars represent 5 *μ*m. (*E*) Given here is a fluorescence signal for subsequent STED microscopy recordings of the same region of interest of CHO cells stained with Di4-ANEPPDHQ (*purple*), Di-4-AN(F)EPPTEA (*blue*), NR12S (*green*), and KK114-DPPE (*red*), relative to the first recording (average and SD from at least five images), indicating sufficient photostability of all probes. (*F*) Given here are representative confocal (*green*) and STED (*red*) microscopy images of two nearby HIV particles membrane-labeled with Di-4-AN(F)EPPTEA and (*G*) intensity profiles along the lines marked in the images. Scale bars represent 200 nm. To see this figure in color, go online.

Next we tested the performance of the polarity-sensitive probes in STED microscopy in terms of spatial resolution and photostability. We again incorporated the probes into CHO cell-derived GPMVs and imaged the equatorial planes with confocal and STED microscopy using 488- and 775-nm laser light for excitation and depletion, respectively. We carried out the experiments on non-phase-separated GPMVs, because we aimed here at imaging the width of the homogeneous membrane. A representative confocal and STED image of the same GPMV stained with Di-4-AN(F)EPPTEA is shown in Fig. 3 *A*. Obviously, the membrane appears thicker in the confocal compared to the STED microscopy image, as highlighted by intensity line profiles across the membrane (Fig. 3 *B*). Because the thickness of the cell membrane (≈8 nm) is well below the expected spatial resolution of both microscopy modes, the thickness of the imaged membrane is a direct indicator of the achieved spatial resolution. To give an estimate of the increase in spatial resolution between confocal and STED microscopy recordings, we determined the width (FWHM) of the respective membrane images using a custom Fiji macro (see Materials and Methods; Fig. S2), and divided the FWHM values obtained for the confocal images by those obtained for the STED microscopy images; values >1 indicate an increase in resolution and the larger the values, the better the improvement. We obtained similar ratios (≈3, 70–90 nm spatial resolution) for all of the tested polarity-sensitive dyes, which were similar to the FWHM values obtained for confocal and STED microscopy images of GPMVs stained with a KK114-labeled phospholipid (Fig. 3 *C*). KK114 (also known as Abberior Star Red) is a well-established dye for STED microscopy (42) and the KK114-stained phospholipid has successfully been employed in previous STED microscopy studies (26, 43). Please note that this relatively modest resolution enhancement, compared to previously published 40–50-nm resolution or 5–6-fold enhancement (35), is primarily due to the depth at which we performed the imaging. Because the equatorial plane of the vesicles were 5–10 *μ*m above the surface, the refractive index mismatch between the oil immersion and the aqueous imaging medium caused aberrations that, in turn, deteriorated the performance of the STED microscope (44).

To confirm the suitability of the polarity-sensitive membrane probes for STED microscopy, we labeled the plasma membrane of live CHO cells with Di-4-AN(F)EPPTEA and imaged it using confocal and STED microscopy. Fig. 3 *D* shows representative side-by-side images in both modes, highlighting the clear improvement in spatial resolution when employing STED microscopy. In addition, time-lapse STED microscopy imaging of the same part of the cells incubated with either of the three fluorescent probes showed reasonable photostability, with <50% (Di-4-ANEPPDHQ), <25% (Di-4-AN(F)EPPTEA and NR12S), and ≈5–10% (KK114-DPPE) loss in fluorescence signal after 20 subsequent images (Fig. 3 *E*).

The efficiency of a STED microscope depends on the fluorescence depletion efficiency by the STED laser light, which roughly scales with the position of the STED laser wavelength at the fluorescence emission spectrum, i.e., the further away the STED laser wavelength from the fluorescence emission maximum, the lower the fluorescence depletion efficiency (21, 22). Therefore, one may expect a worse performance of the polarity-sensitive dyes in STED microscopy when incorporated in more ordered membrane environments, where the emission spectrum is blue shifted. To test this, we next explored the possible use of the polarity-sensitive dyes for imaging and separating subdiffraction-sized HIV particles. The HIV membrane environment exhibits a very high membrane order, much larger than in the cellular plasma membrane (45). We labeled HIV particles (≈100–150 nm in diameter) with Di-4-AN(F)EPPTEA and imaged them, after immobilization on a poly-L-lysine-coated microscope cover glass. Fig. 3, *F* and *G*, highlights the separation of nearby virus particles in STED microscopy imaging but not in the confocal mode, despite the more blue-shifted spectra.

**Figure 4:**
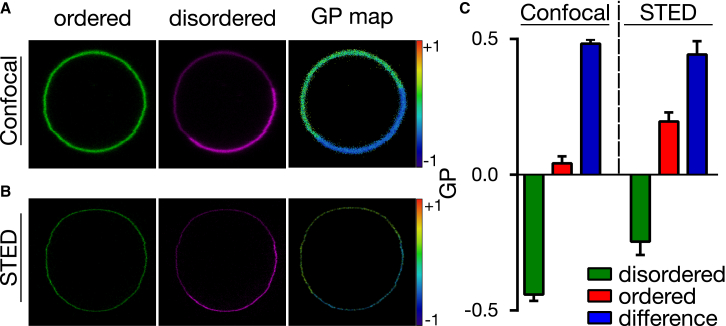
STED GP imaging of micronscale phase-separated GPMVs prepared using high DTT concentration (30 mM) and labeled with Di-4-AN(F)EPPTEA. (*A* and *B*) Shown here are confocal (*A*) and STED (*B*) microscopy images (20 × 20 *μ*m; *green*, 520–570 nm, ordered channel; *magenta*, 650–700 nm, disordered channel) of a phase-separated GPMV and (*right*) GP map created therefrom. Red-to-blue color code: GP values from maximally ordered (*red*, +1) to maximally disordered (*blue*, −1). (*C*) Given here are the average and SD (error bars) of GP values determined from the ordered (*red*) and disordered (*green*) phases of at least 10 GPMVs imaged in confocal and STED mode, and respective differences in GP values between the phases (*blue*). To see this figure in color, go online.

Next, we imaged spatial heterogeneity in molecular order of the biomembranes using superresolution STED microscopy. To this end, we employed GP as an index commonly used to quantify the differences in polarity of the membrane environment by exploiting the spectral shift of the polarity-sensitive dyes and to relatively compare molecular membrane ordering (or lipid packing; see Materials and Methods for the details on calculation of GP maps). Confocal GP imaging is a common tool for highlighting the spatial heterogeneity in the membranes (38). Thus, we tested whether GP can be measured using the selected polarity-sensitive dyes in combination with STED microscopy. Therefore, we prepared CHO cell-derived GPMVs that exhibited microscopic phase separation at room temperature (using 30 mM DTT) (46). We labeled the phase-separated GPMVs with Di-4-AN(F)EPPTEA and imaged the fluorescence emission in two separate spectral channels (520–570 and 620–700 nm for ordered and disordered membrane environments, respectively) both in confocal (Fig. 4 *A*) and STED mode (Fig. 4 *B*). We later calculated GP values for each image pixel using our previously published GP plugin to create the respective GP maps (38). The STED GP images of the phase-separated GPMVs were similar to their confocal counterparts, yet with a significantly improved spatial resolution. The relative interdomain GP difference was similar for both confocal and STED microscopy recordings (*blue bars* in Fig. 4 *C*), yet with slightly higher absolute GP values in STED mode (*green* and *red bars* in Fig. 4 *C*). This is to be expected, because the red-shifted fluorescence emission in the higher wavelength channel (disordered environment) is closer to the wavelength of the STED laser and thus more efficiently inhibited by stimulated emission. This results in a relatively higher decrease in fluorescence emission in the higher wavelength channel compared to the lower wavelength channel and consequently yields higher absolute GP values. However, it is worth noting that GP is a relative parameter; the absolute values vary dramatically depending on the experimental conditions, the probes, or the microscopes (38). Thus, the relative change in GP (such as the interdomain GP difference) is more indicative. Similar relative change in GP values between ordered and disordered environments in the confocal and the STED modes confirms the accuracy and applicability of STED GP imaging. Here, it is also important to note that the shift in emission wavelengths due to the lipid packing might result in variable spatial resolutions while imaging more ordered (lower emission wavelengths) and more disordered (higher emission wavelengths) phases, which may further bias STED GP imaging. However, we obtained similar spatial resolutions for ordered and disordered phases in GPMVs in the lower and the higher wavelength channels (Fig. S5) that further renders the STED GP imaging applicable.

**Figure 5:**
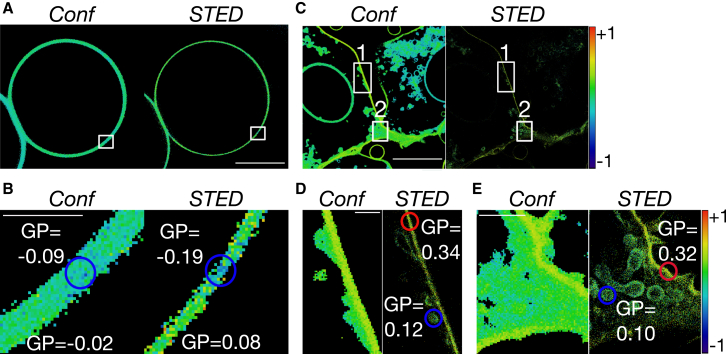
STED GP imaging of membrane nanostructures. (*A* and *B*) Given here are confocal and STED GP images of the equatorial planes of a CHO-derived GPMV prepared with 5 mM DTT and labeled with Di-4-AN(F)EPPTEA: (*A*) overviews (scale bars, 10 *μ*m) and (*B*) closeups into the white squares (scale bars, 0.5 *μ*m), highlighting the improved GP determination of a nanodomain of low molecular order (*blue circle*) in the STED mode (*GP* = −0.19 ± 0.05 (average ± SD) and −0.09 ± 0.07 for the domain compared to *GP* = 0.08 ± 0.03 and −0.02 ± 0.05 for the surrounding membrane environment in the STED and confocal modes, respectively). (*C*–*E*) Given are the confocal and STED GP images of the plasma membrane of live CHO cells labeled with Di-4-AN(F)EPPTEA: (*C*) overviews (scale bars, 10 *μ*m) and (*D* and *E*) closeups into the marked areas (*rectangle* 1 (*D*) and *rectangle* 2 (*E*); scale bars, 0.5 *μ*m), highlighting the improved GP determination of internal vesicles of low molecular order (*blue circles*) in the STED recordings (*GP* = 0.11 ± 0.09 for the vesicles compared to *GP* = 0.32 ± 0.07 of the plasma membrane (*red circles*)). Red-to-blue color code: GP values from maximally ordered (*red*, +1) to maximally disordered (*blue*, −1). To see this figure in color, go online.

Next, we performed STED GP imaging of cellular membrane nanostructures. We first prepared GPMVs exhibiting smaller domains (47) (using 5 mM DTT; see Materials and Methods for details). We did not observe any obvious microscopic phase separation in these GPMVs in confocal mode, but instead homogeneous GP values ranging between 0 and −0.1 (Fig. 5 *A*). In the closeup confocal GP image (Fig. 5 *B*), an indication of a domain was present, yet it was quite unclear and GP determination was challenging. However, the corresponding STED GP map clearly revealed a small domain of lower lipid order (*GP* = −0.19) than the surrounding membrane environment (*GP* = 0.08) (Fig. 5 *B*).

Besides membrane nanodomains, we used STED GP imaging to investigate small (∼100–200 nm) internal endocytic vesicles located close to the plasma membrane, whose lipid packing is challenging to determine in the conventional confocal GP images due to the limited spatial resolution of confocal microscopy. We stained live CHO cells with Di-4-AN(F)EPPTEA for 15 min at 37°C during which the dye partially entered the cells, staining internal structures such as endocytic vesicles. These structures were imaged using confocal and STED microscopy and GP images were generated therefrom as before (Fig. 5 *C*). Closeups into the STED GP images clearly highlighted the presence of such vesicles, which remained unresolved in the confocal counterparts (Fig. 5, *D* and *E*). Most importantly, the improved resolution of the STED mode now allowed us to determine the lipid packing of these vesicles and to compare it to the lipid packing of the plasma membrane. Obviously, the vesicular membranes had a significantly lower molecular order (*GP* ∼ 0.1) than the plasma membrane (*GP* > 0.3), which is in line with predictions from previous measurements (48).

## Conclusions

We have shown the compatibility of the polarity-sensitive membrane dyes Di-4-ANEPPDHQ, Di-4-AN(F)EPPTEA, and NR12S for superresolution STED microscopy. Using cell-derived GPMVs, live CHO cells, and HIV particles, we highlighted for all three probes 1) a sufficient sensitivity for distinguishing disordered and ordered environments in cellular membranes, 2) the possibility to spectrally separate their fluorescence signal from GFP signal, 3) the successful application in superresolution STED microscopy imaging of cellular membranes, and 4) a sufficiently large photostability allowing for time-lapse imaging of living cells. Moreover, we successfully demonstrated that spectrally resolved STED imaging is possible and differences in lipid packing of the membranes on the nanoscale can be inferred from these images. We exemplified this by showing the differences in molecular order of microscopic or nanoscopic domains in GPMVs, and of small internal vesicles in living cells. These features provide a starting point for future measurements of lateral heterogeneity in lipid order in the plasma and internal membranes of living cells, also in relation to the organization of, for example, GFP-labeled proteins. This may shed new light on the existence and function of possible lipid nanodomains or clusters of different molecular order. Whereas these domains are expected to be even smaller than the spatial resolution achieved so far in this work, we expect, with further optimization of the setup (such as laser intensity and wavelength and corrections for optical aberrations) and the fluorophores, to improve the resolution even further.

For accurate determination of lipid packing with STED GP imaging, there are certain points to consider. First, the spatial resolution should be similar for both the lower and the higher wavelength channels. Unfortunately, this might not be the case when membrane environments with significantly different lipid packing are imaged. Due to the spectral shift, the STED depletion efficiency in two channels might be different. Although we did not observe a notable difference in spatial resolution for phase-separated GPMVs, such differences in resolution may appear in the case of larger differences in lipid packing and thus spectral shift, as in artificial membranes such as phase-separated giant unilamellar vehicles. Using a spectral detector for STED GP imaging (instead of the detection in two spectrally separated channels as done here) will help us to better control the resolution and also improve the spectral sensitivity, giving the potential to directly observe membrane nanodomains of different lipid order.

Another point to consider is the image acquisition time. In our current setup, the recording of a superresolved GP image takes a few seconds, which is well above the timescales of dynamics in cellular membranes. We expect to increase the temporal resolution further through improved detection and beam-scanning, thus guiding the way toward improved investigations of membrane dynamics.

Overall, this study shows that STED imaging with polarity-sensitive dyes is a useful tool to investigate the cell membrane heterogeneity. It also sets a guideline for the development of future polarity-sensitive probes suitable for advanced superresolution imaging.

## Author Contributions

E.S., F.S., I.U., and V.Z. carried out experiments. E.G. helped with microscopy recordings. D.W. wrote the analysis software and helped with analyzing the data. A.S.K. supplied reagents. E.S. and C.E. conceived the study and wrote the manuscript with help from all authors. All authors contributed in discussing the data, experiments, and the manuscript.

## Supporting Material

Document S1. Figs. S1–S5

Document S2. Article plus Supporting Material
