# Supplementary material for: Polarity-Sensitive Probes for Superresolution Stimulated Emission Depletion Microscopy
Source: Biophys J. 2017 Jul 19;113(6):1321–30. doi: 10.1016/j.bpj.2017.06.050 (PMC5607142; doi:10.1016/j.bpj.2017.06.050)
Supplement: Document S1. Figs. S1–S5 [file mmc1.pdf]

Biophysical Journal, Volume *113*

Supplemental Information

Polarity-Sensitive Probes for Superresolution Stimulated Emission Depletion Microscopy

Erdinc Sezgin, Falk Schneider, Victoria Zilles, Iztok Urbančič, Esther Garcia, Dominic Waithe, Andrey S. Klymchenko, and Christian Eggeling

Supplementary Figures

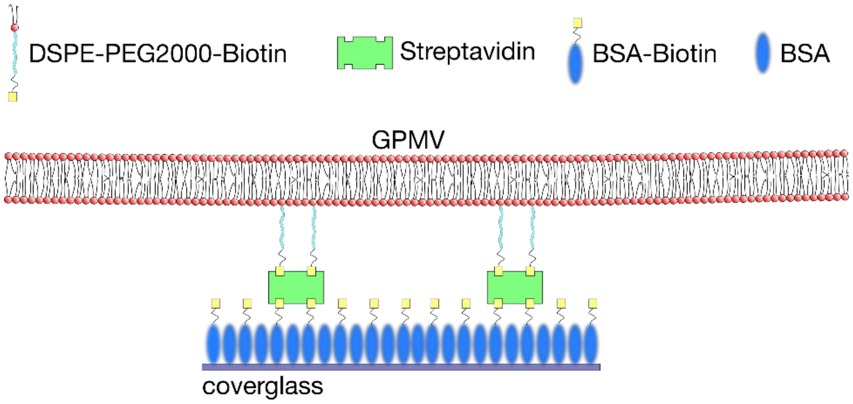

Supplementary Figure S1. Immobilisation protocol of GPMVs. Microscope cover glass was coated with BSA and BSA-biotin, and Streptavidin was added. GPMVs doped with DSPE-PEG200-Biotin lipids bound to the cover glass through the Streptavidin-biotin interaction.

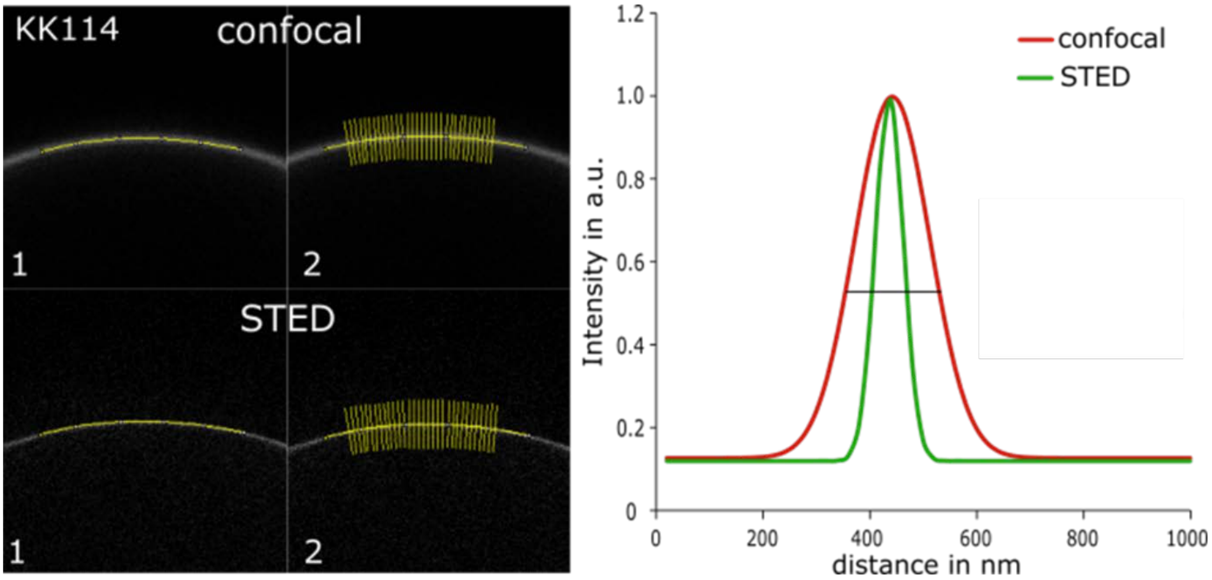

Supplementary Figure S2. FiJi plugin for the determination of the width (FWHM) of the intensity profiles across the membranes of GPMVs as imaged with confocal or STED microscopy. (Left) Representative section of a confocal (upper) and STED (lower) microscopy image of the equatorial plane of a giant-unilamellar-vesicle (GUV, 100 % DOPC) stained with KK114-DPPE (0.1 mol% KK114-DPPE). First (panel 1), a segmented line (yellow) is drawn along the membrane, followed by (panel 2) the determination of an intensity profile along the line (yellow) perpendicular to the previously segmented line for every third pixel. (Right) Exemplary line profiles (red: confocal, and green: STED microscopy recordings) obtained in this way, allowing for the determination of FWHM values (black line).

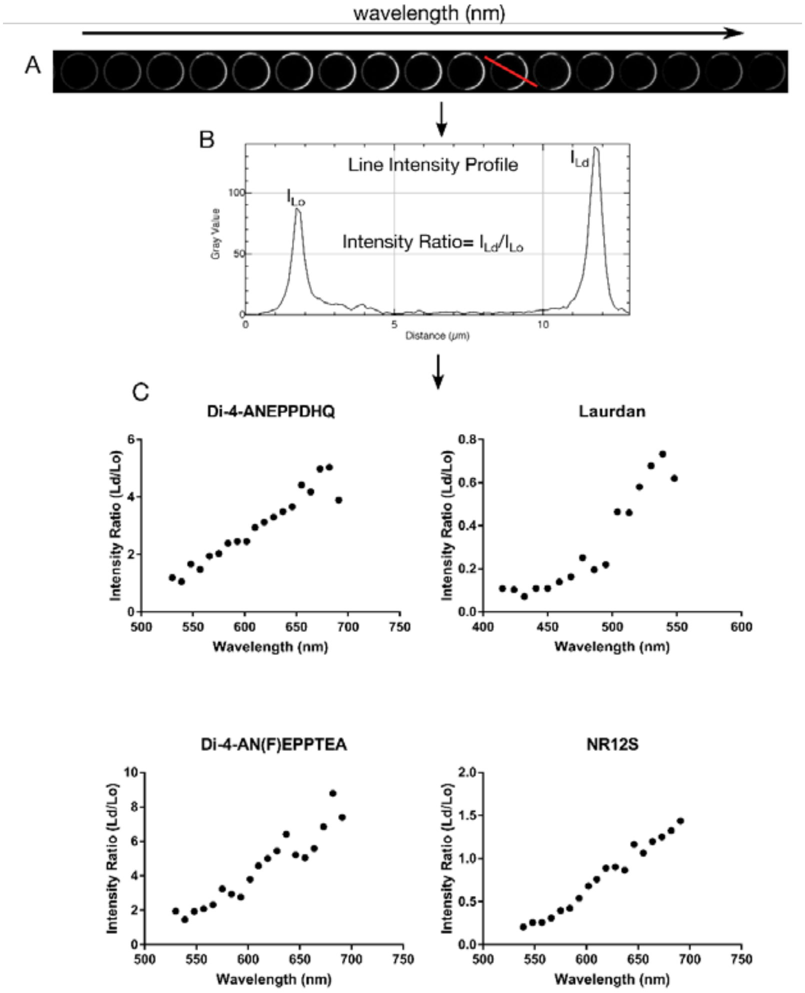

Supplementary Figure S3. Wavelength dependence of the fluorescence emission intensity of the polarity sensitive probes in ordered and disordered phases of CHO cell-derived GPMVs. A) Representative spectrally resolved confocal images of the equatorial plane of a phase-separated GPMV stained with Di-4-AN(F)EPPTEA (20 µm × 20 µm). B) Representative intensity profile along the line crossing membrane segments with different phases as indicated by the red line in panel (A), allowing for the determination of the ratio I_{Ld}/I_{Lo} of fluorescence intensities detected for the disordered (Ld, I_{Ld}) and ordered (Lo, I_{Lo}) membrane phases. C) Exemplary wavelength dependence of the intensity ratios determined from intensity line profiles from phase-separated GPMVs (as shown in panel (B)) for Laurdan, Di-4-ANEPPDHQ, Di-4-AN(F)EPPTEA and NR12S as marked. The values of intensity ratios increased for higher wavelengths due to the common red-shift in fluorescence emission in the more disordered Ld phase. One would expect values of I_{Ld}/I_{Lo} close to 0 at very low wavelengths (were $I_{Ld} \approx 0$) and >> 1 at large wavelengths (where $I_{L0} \approx 0$). Deviations thereof indicate changes in the overall emission characteristics of the dyes in the different membrane environments, e.g. due to changes in the fluorescence quantum yield. The overall fluorescence emission was higher (> 1 at all wavelengths) in the disordered phases for Di-4-ANEPPDHQ and Di-4-AN(F)EPPTEA, lower (<1 at all wavelengths) for Laurdan, and changing with wavelength (ratios <1 for lower and >1 for higher wavelengths) for NR12S.

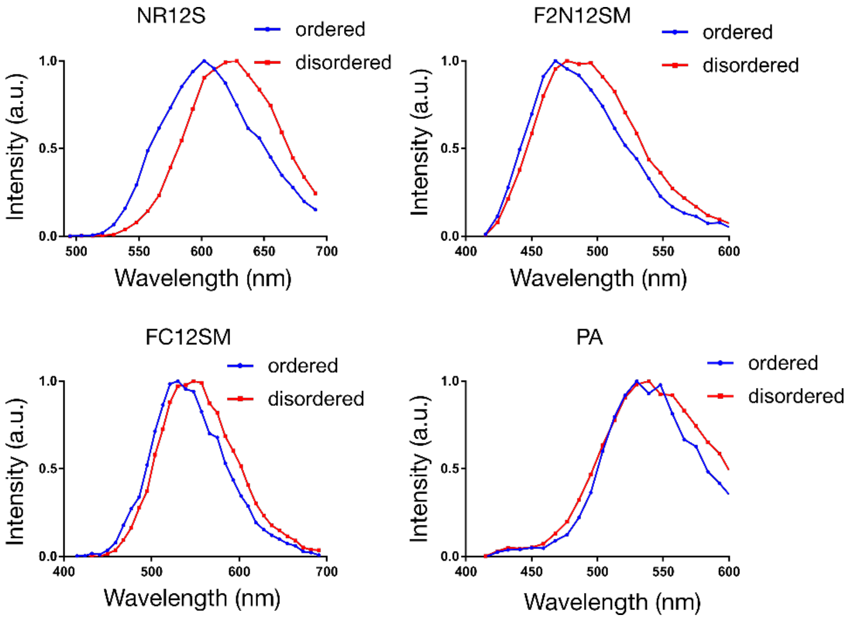

Supplementary Figure S4. Fluorescence emission spectra of NR12S (same data as in Fig. 2E in the main text, repeated here for comparison), F2N12SM, FC12SM and PA as averaged over the Ld (red) and Lo (blue) phases of the confocal images of the equatorial plane of accordingly-stained phase-separated GPMVs, indicating a lower spectral shift between ordered and disordered membrane environment for F2N12SM, FC12SM and PA.

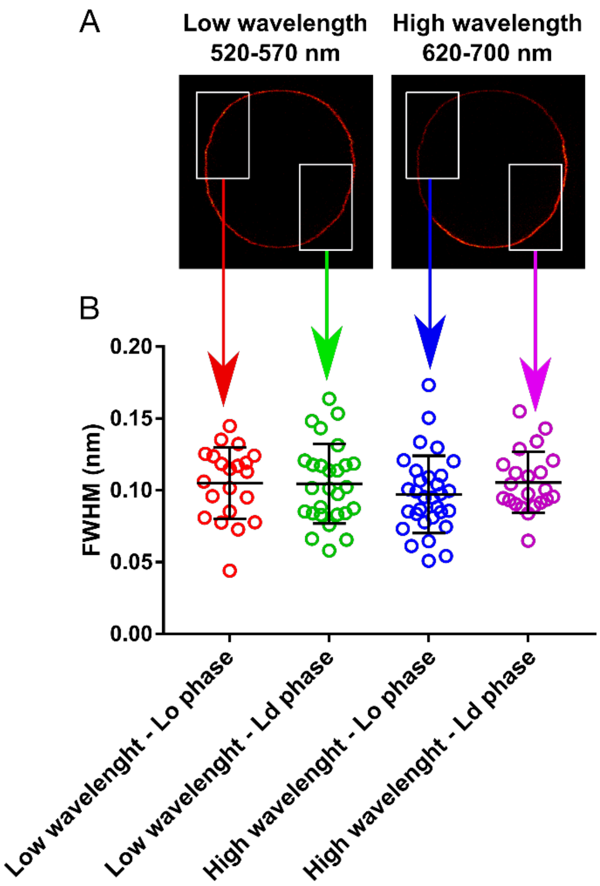

Supplementary Figure S5. Spatial resolution in the STED images of different channels and phases. A) Representative STED images of the equatorial plane of phase-separated GPMVs for the lower-wavelength (left) and upper-wavelength (right) detection range. B) According values of the width (FWHM) of the intensity profiles across the membranes for the respective ordered and disordered phases, indicating no significant change in spatial resolution for the different wavelength ranges and phases.
